# Supplementary material for: A Novel Association between YKL-40, a Marker of Structural Lung Disease, and Short Telomere Length in 10-Year-Old Children with Bronchopulmonary Dysplasia
Source: Children (Basel). 2021 Jan 24;8(2):80. doi: 10.3390/children8020080 (PMC7912154; doi:10.3390/children8020080)
Supplement: Supplementary file 1 [file children-08-00080-s001.pdf]

Supplementary material.

Table S1. Cohort characteristics, neonatal morbidity and markers of lung disease.

	**BPD/preterm All n = 29**	**BPD/preterm with SPECT n = 25**	**Asthma/term n = 28**
Clinical characteristics			
birth weight, grams	1070 ± 423**	991 ± 340	3591 ± 356**
age at birth, weeks	27^{+1} ± 2.4**	26^{+2} ± 2.3	40^{+0} ± 1.3**
cesarian section, n (%)	17 (59) **	15 (60)	5 (25)[b] **
sex, male (%)	20 (69)	18 (72)	19 (68)
maternal age, years	32 ± 4	32 ± 4	32 ± 4
paternal age, years	34 ± 6	33 ± 5	34 ± 7
age at study follow-up, years	10.4 ± 0.9	10.4 ± 1.0	10.6 ± 0.6
female	11.0 ± 1.1*	11.2 ± 1.2	10.7 ± 0.7
male	10.1 ± 0.6*	10.1 ± 0.7	10.6 ± 0.6
Neonatal morbidity			
supplementary oxygen, days	76 ± 38	81 ± 12	
CPAP, days	38 ± 16	40 ± 16	
ventilator, days	10 ± 12	11 ± 13	
antenatal steroids, n (%)	23 (85)[a]	19 (82)[a]	
surfactant ≥ 1 dose, n (%)	18 (69)[b]	16 (73)[b]	
treated PDA, n (%)	19 (66)	16 (64)	
moderate/severe BPD, n (%)	16 (55)	15 (60)	
Lung function at follow-up age 10			
FEV_1, %	77 ± 12*	76 ± 12	84 ± 11*
FEF_{50}, %	61± 16**	61 ± 16	79 ± 21**
FEF_{25-75}, %	55 ± 14**	54 ± 14	69 ± 19**
DLCO, mmol/min/kPa	81 ± 13*	80 ± 12	88 ± 10*
FENO, ppb	12 ± 9*	12 ± 10	23 ± 21*
RTL	1.56 [0.3]	1.56 [0.3]	1.52 [0.4]
YKL-40	17.7 ± 6.3**	17.2 ± 6.1	13.2 ± 3.8**

Mean ± SD or median [IQR]; *significance $p < 0.05$; **significance $p < 0.01$; [a]missing value (n = 2); [b]missing value, (n = 3); [c]missing value (n = 4); Dynamic spirometry values are reported as percentages of expected values corrected for length; FEV_1 = forced expiratory volume in one second; FEF_{25-75} = forced expiratory volume , 25-75%; DLCO = diffusion capacity of carbon monoxide reported as percentage of expected values adjusted for age; FENO = fractional exhaled nitric oxide; ppb = parts per billion, PDA = patent ductus arteriosus; ROP = retinopathy of prematurity; CPAP = continuous positive airway pressure; RTL = relative telomere length; YKL-40 = chitinase-3-like protein 1

Table S2. Associations between the relative telomere length (RTL) and YKL-40 and spirometry at ten years of age in children born preterm with a history of BPD.

**Lung function**	**YKL-40** *Coefficient (95% CI*
FEV_1	-0.07 [-0.27; 0.14]
FVC	-0.11 [-0.28; 0.07]
FEF_{25}	-0.02 [-0.18; 0.14]
FEF_{50}	-0.05 [-0.21; 0.10]
FEF_{75}	-0.01 [-0.17; 0.16]
FEF_{25-75}	-0.02 [-0.23; 0.20]
DLCO, mmol/min/kPa	-0.02 [-0.23; 0.20]

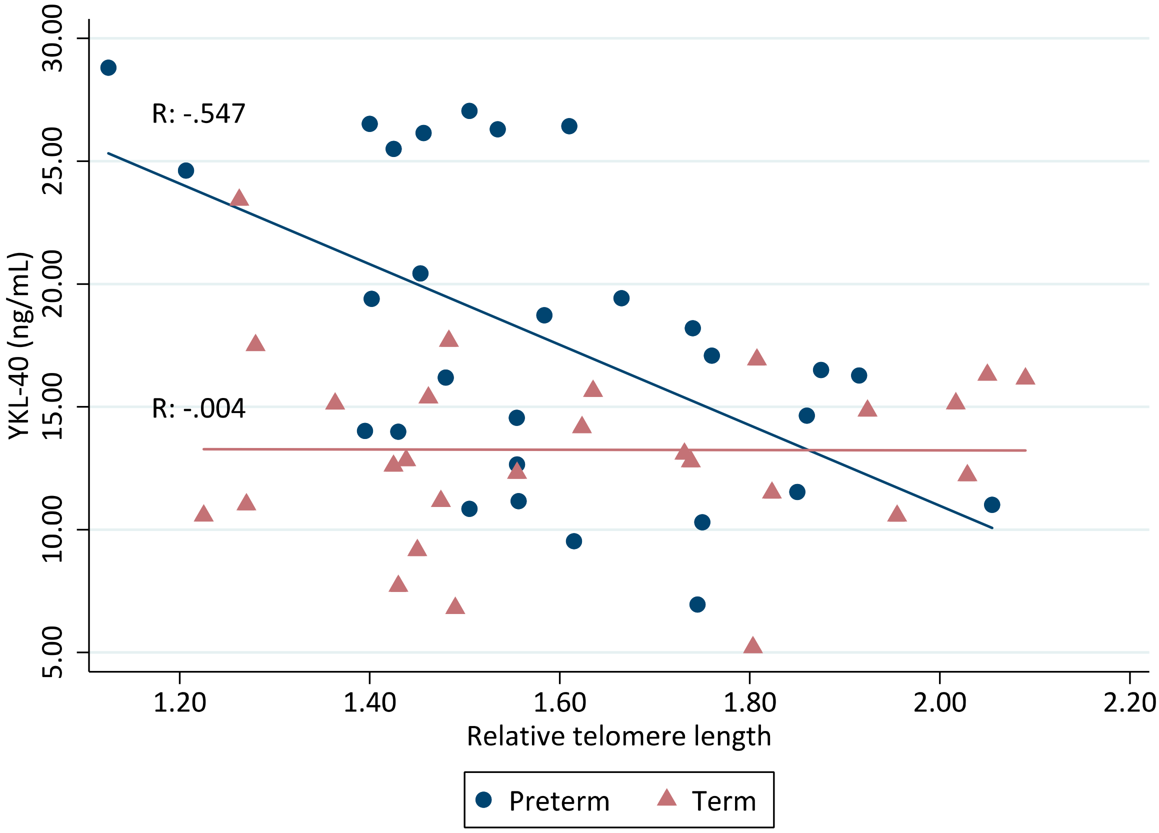

Figure S1. Correlations between relative telomere length (RTL) and YKL-40 at ten years of age. Children born preterm with a history of bronchopulmonary dysplasia ($n = 29$) showed significantly higher levels of YKL-40 correlating to shorter RTLs (<0.01) compared to children born term with allergic asthma ($n = 28$)($p > 0.05$).
